# SARS-CoV-2 bivalent mRNA vaccine with broad protection against variants of concern

**DOI:** 10.3389/fimmu.2023.1195299

**Published:** 2023-05-24

**Authors:** Qinhai Ma, Man Li, Lin Ma, Caroline Zhang, Hong Zhang, Huiling Zhong, Jian Wen, Yongsheng Wang, Zewei Yan, Wei Xiong, Linping Wu, Jianmin Guo, Wei Yang, Zifeng Yang, Biliang Zhang

**Affiliations:** ^1^ State Key Laboratory of Respiratory Disease, National Clinical Research Center for Respiratory Disease, Guangzhou Institute of Respiratory Health, the First Affiliated Hospital of Guangzhou Medical University, Guangzhou, China; ^2^ Department of Drug Discovery and Development, Argorna Pharmaceuticals Co., Ltd, Guangzhou, China; ^3^ Department of Manufacturing, Guangzhou RiboBio Co., Ltd, Guangzhou, China; ^4^ Laboratory of Computational Biomedicine, Guangzhou Institutes of Biomedicine and Health, Chinese Academy of Sciences, Guangzhou, China; ^5^ Guangdong Provincial Key Laboratory of Drug Non-clinical Evaluation and Research, Guangdong Lewwin Pharmaceutical Research Institute Co., Ltd., Guangzhou, China; ^6^ State Key Laboratory of Quality Research in Chinese Medicine, Macau University of Science and Technology, Macau, Macau SAR, China

**Keywords:** SARS-CoV-2, bivalent mRNA vaccine, variants of concern (VOCs), immunogenicity, broad-spectrum efficacy

## Abstract

**Introduction:**

The severe acute respiratory syndrome coronavirus 2 (SARS-CoV-2) Omicron variant has rapidly spread around the globe. With a substantial number of mutations in its Spike protein, the SARS-CoV-2 Omicron variant is prone to immune evasion and led to the reduced efficacy of approved vaccines. Thus, emerging variants have brought new challenges to the prevention of COVID-19 and updated vaccines are urgently needed to provide better protection against the Omicron variant or other highly mutated variants.

**Materials and methods:**

Here, we developed a novel bivalent mRNA vaccine, RBMRNA-405, comprising a 1:1 mix of mRNAs encoding both Delta-derived and Omicron-derived Spike proteins. We evaluated the immunogenicity of RBMRNA-405 in BALB/c mice and compared the antibody response and prophylactic efficacy induced by monovalent Delta or Omicron-specific vaccine with the bivalent RBMRNA-405 vaccine in the SARSCoV-2 variant challenge.

**Results:**

Results showed that the RBMRNA-405 vaccine could generate broader neutralizing antibody responses against both Wuhan-Hu-1 and other SARS-CoV-2 variants, including Delta, Omicron, Alpha, Beta, and Gamma. RBMRNA-405 efficiently blocked infectious viral replication and lung injury in both Omicron- and Delta-challenged K18-ACE2 mice.

**Conclusion:**

Our data suggest that RBMRNA-405 is a promising bivalent SARS-CoV-2 vaccine with broad-spectrum efficacy for further clinical development.

## 1 Introduction

The outbreak of the coronavirus disease 2019 (COVID-19) pandemic led to the rapid development and approval of various vaccines around the world in efforts to control the spread of the virus. However, severe acute respiratory syndrome coronavirus 2 (SARS-CoV-2) continues to mutate, leading to over 500 million confirmed cases with more than 6 million deaths in the past 2 years (https://covid19.who.int/). Since the World Health Organization (WHO) declared a global pandemic in 2020, more than eight variants of SARS-CoV-2 have emerged over the globe including the Alpha (B.1.1.7), Beta (B.1.351), and Gamma (P.1) variants (1). These emerging variants of concern (VOCs) were reported to have resistance against neutralizing antibodies (NAbs) and increased breakthrough infections, and are more infectious (2–4). Messenger RNA (mRNA) vaccines have emerged as ideal platforms for rapid vaccine design, and they can be developed based on sequence information alone without the need for virus culture (inactivated or live attenuated vaccines) or scaled recombinant protein production. In addition, mRNA vaccines avoid the problem of insertional mutagenesis of DNA-based vaccines and pre-existing immunity that can dampen immunogenicity of viral vector vaccines. Recently, various mRNA vaccine platforms have been developed in recent years and validated in studies of immunogenicity and efficacy (5, 6).

The Omicron (B.1.1.529) variant was first described by South Africa and Botswana on 24 November 2021 and swiftly became the predominantly circulating strain in most countries (7). This variant contains at least 15 mutations located in the spike (S) receptor-binding domain (RBD) (8), which is the main antigen target for NAbs (9). Compared to other VOCs, the Omicron variant exhibits significant immune evasion due to the large number of mutations in its S protein that was not observed in the ancestral strain (also referred to as wild-type, Wuhan-Hu-1) or previous variants. The impact of SARS-CoV-2 variants on existing vaccines was assessed by a great number of studies (10–15), which show dramatic reduction in protection against the Omicron variant. Therefore, a recommended third-dose booster vaccination has become a commonly used strategy to augment the potency and duration of anamnestic responses against the emerging VOCs (16–19). Although current vaccines have largely been effective against past variants, those who had received three doses of COVID-19 vaccines, including at least two doses of an mRNA vaccine, remained to experience breakthrough infections, characterizing the consequences of the highly mutated RBD seen in Omicron (4). Thus, emerging VOCs have brought new challenges to the prevention of COVID-19 and updated vaccines are urgently needed to provide better protection against the Omicron variant or other highly mutated variants.

mRNA vaccines are considered as a promising vaccine platform due to its safety, versatility, rapid development process, comparatively low production cost, and higher efficacy (5). The approved mRNA vaccines, BNT162b2 (Pfizer/BioNTech) and mRNA-1273 (Moderna) designed against the Wuhan-hu-1 strain (WA1/2020, WT) of SARS-CoV-2, were authorized for emergency use in 2020 and attained vast success in limiting viral spread and lowering hospitalization and death risks. Effectiveness of these mRNA vaccines has been investigated extensively following their mass rollout. The effectiveness of two doses of the aforementioned vaccines for symptomatic infection was above 90% (20). Two doses of BNT162b2 have estimated effectiveness of 87% for hospitalization and 92% for severe COVID-19 (21). Previously, Ma et al. developed a lipid nanoparticle (LNP)-encapsulated mRNA vaccine candidate, RBMRNA-176, a pseudouridine nucleoside-modified mRNA vaccine that encodes prefusion stabilized trimeric S protein ectodomain, which have been shown effective in neutralizing SARS-CoV-2 in mice and nonhuman primates (22). Considering that RBMRNA-176 showed significant reduction in neutralization antibody titers against Omicron (22), an updated mRNA vaccine that directly targets the heavily mutated S protein of Omicron was deemed critical. We therefore developed a novel bivalent mRNA vaccine candidate termed RBMRNA-405 against SARS-CoV-2 Delta and Omicron variants based on our established LNP-mRNA vaccine platform. Here, we assessed the immunogenicity and antiviral protective effect of RBMRNA-405 in challenge studies. Following the vaccination of RBMRNA-405 in mice, we found that the vaccine candidate induced robust Th1-biased T-cell responses and potent germinal center (GC) B-cell responses, including elevated B cells, GC B cells, memory B cells (MBCs), and plasma cells (PCs), as well as T follicular helper (T_fh_) cell responses. Notably, RBMRNA-405 induced NAbs with potential cross-neutralizing activity against Delta and Omicron variants. Vaccinated K18-hACE2 transgenic mice exhibited little-to-no viral-induced pathology in the lung and spleen tissue following lethal challenge with Delta and Omicron variants. Taken together, our data suggest RBMRNA-405 as a promising bivalent SARS-CoV-2 vaccine with broad-spectrum efficacy for further clinical development.

## 2 Methods

### 2.1 Materials availability

All requests for unique/stable reagents generated in this study should be directed to and will be fulfilled by the lead contact author with a completed material transfer agreement.

### 2.2 Data and code availability

All the other data supporting the finding of this study are available within the paper and are available from the corresponding author upon request. This study did not generate unique code.

### 2.3 Ethics statement

BALB/c mice (6–8 weeks) were obtained from Zhejiang Vital River Laboratory Animal Technology Co., Ltd. and housed in SPF Laboratory Animal Facility under a 12-h light/dark cycle, with free access to food and water. BALB/c mouse experiments were performed in strict accordance with the requirements of Guangdong experimental animal management regulations and Institutional Animal Care and Use Committee (IACUC Approval No.: IACUC-2021-026). K18-hACE2 (6–9 weeks) transgenic mice were used and the experiments were performed in accordance with protocols approved by the Animal Care and Use Committee of Guangzhou Medical University (Acceptance number: 2018-297). All work with live SARS-CoV-2 virus was performed in Biosafety Level 3 (BSL-3) containment laboratories.

### 2.4 mRNA synthesis and formulation

A DNA fragment encoding the full-length spike sequence of the Omicron variant (lineage B.1.1.529/BA.1, Genbank: OM287553.1) or Delta variant (lineage B.1.617.2, Genbank: ON220436.1) was cloned into a starting plasmid vector with non-coding backbone elements for improved RNA stability and translational efficiency, including the regions from the T7 promoter to the 5’ and 3’ untranslated regions (UTRs) (23, 24), and a 120-nt poly-A tail (25). The sequence was human codon optimization and synthesized *in vitro* by T7 polymerase-mediated transcription where the uridine-5′-triphosphate (UTP) was substituted with pseudouridine-5’-triphosphate (pseudo-UTP). Capped mRNAs were generated by supplementing the transcription reactions with RIBO-Cap4. mRNA was purified by reversed-phase high-performance liquid chromatography (RP-HPLC) (26). RNA quality was analyzed by bioanalyzer analysis (Agilent 2200 Tape station). mRNA concentrations were measured by UV spectroscopy.

The preclinical mRNA vaccines used in this study include the following: (1) monovalent RBMRNA-Delta vaccine that contains a single mRNA encoding SARS-CoV-2 spike antigen of Delta; (2) monovalent RBMRNA-404 vaccine that contains a single mRNA encoding SARS-CoV-2 spike antigen of Omicron; (3) bivalent RBMRNA-405 vaccine, which is a 1:1 mix of separately formulated RBMRNA-Delta and RBMRNA-404 in a vial. Each mRNA is separately formulated into LNPs and then mixed, prior to vialing so both mRNA formulations are present in the vial. Lipid nanoparticles were prepared by microfluidic mixing using the previously described method (27). Briefly, lipids were dissolved in ethanol at molar ratios of 45:16:15:1.0 (ionizable lipid:cholesterol:DSPC : DMG-PEG2000). The lipid mixture was rapidly combined with a buffer of 50 mM sodium citrate (pH 4.0) containing mRNA at a volume ratio of aqueous:ethanol using a microfluidic mixer (PNI Nanosystems, Vancouver, BC, Canada). Formulations were dialyzed against PBS (pH 7.2) in the dialysis cassettes (Thermo Scientific, Rockford, IL, USA) for at least 18 h. Formulations were diluted with PBS (pH 7.2) to reach a required concentration, and then passed through a 0.22-mm filter and stored at 4˚C until use. Formulations were analyzed for particle size, mRNA encapsulation, residues, endotoxin, and bioburdens (28), and they were found to be sterile, between 80 and 125 nm in size, with≥90% encapsulation and ≤2 EU/ml endotoxin.

### 2.5 Animals and immunizations

Immunization of mice was performed following a similar approach that was previously reported (28). Briefly, female BALB/c mice (6–8 weeks) were randomly divided into groups of *n* = 6 or 3. The mRNA vaccines were diluted in PBS and injected I.M. into the gastrocnemius muscle at three doses (1 μg, 5 μg, and 20 μg/dose), respectively. All grouped animals were immunized on Day 0 and received a booster vaccination on Day 21 (28). The control group was injected with PBS. For sequential immunization, female BALB/c mice (6–8 weeks, *n* = 6 or 3 per group) were I.M. injected with two doses of 3 μg of inactivated SARS-CoV-2 vaccine (CoronaVac, Sinovac) on Week 0 and Week 3, and 24 weeks after the second vaccine dose, mice were boosted with 3-μg doses of inactivated vaccine or 20-μg doses of RBMRNA-405. The control group was injected with PBS buffer.

K18-hACE2 mice used for SARS-CoV-2 variants challenge experiment were randomly divided into seven groups of *n* = 5. Three of the seven groups were used for the Delta variant challenge, and another three groups were used for the Omicron variant challenge. The remaining group was used for the mock challenge. All grouped animals were injected I.M. into the gastrocnemius muscle twice with 5- or 20-μg doses of RBMRNA-404 or RBMRNA-405 on Day 0 and Day 21. The control group and mock challenge group were injected with physiological saline.

### 2.6 Enzyme-linked immunosorbent assay

To determine serum total IgG, IgG1, and IgG2a, recombinant Delta/SARS-CoV-2 Spike Trimer protein (ACROBiosystems, SPN-C52He), recombinant B.1.1.529/Omicron SARS-CoV-2 Spike Trimer protein (ACROBiosystems, SPN-C52Hz), and recombinant wild type SARS-CoV-2 Spike Trimer protein (ACROBiosystems, SPN-C52H4) were diluted to 2 μg/ml, respectively, in carbonate (0.05 M, pH 9.6). Ninety-six-well plates (JET BIOFIL) were coated with the diluted antigen (200 ng/well) and incubated at 4°C overnight. The plates were washed with PBS-T (0.05% Tween-20) three times and were blocked with 1% BSA in PBS for 1–2 h at 37°C. Then, the plates were washed and heat-inactivated serum serially diluted in PBST. Diluted serum samples (100 μl/well) were added and incubated for 1 h at 37°C. After washing the plates three times with PBS-T, HRP-conjugated Goat anti-mouse IgG (H+L) (Proteintech, SA00001-1, 1:1,000), HRP-conjugated Goat anti-Mouse IgG1 (Bethyl, A90-105P, 1:5,000), or HRP-conjugated Goat anti-Mouse IgG2a (Bethyl, A90-107P, 1:5,000) was added and incubated for 1 h at 37°C. Plates were then washed and 100 μl of TMB peroxidase substrate (TIANGEN, PA107-02) was added to each well for development. After 15 min, plate development was halted by the addition of 2 mol H_2_SO_4_ and the absorbance was read at 450 nm using TECAN infinite M200 pro. Endpoint titers were calculated as the highest serum dilution that exceed the cutoff values (mean ± SD of negative controls at the lowest dilution).

### 2.7 ELIspot

Mouse ELIspot assays were performed with Mouse IL-4 precoated ELISPOT kit (Dakewe Biotech, 2210402), Mouse IL-5 ELIspotPLUS (HRP) (Mabtech, 3391-4HPW-2), Mouse IL-2 ELIspotPLUS (HRP) (Mabtech, 3441-4HPW-2), and Mouse IFN-γ precoated ELISPOT kit (Dakewe Biotech, 2210005) according to the manufacturer’s instructions. Briefly, the plates were blocked using RPMI 1640 (GIBCO) containing 10% fetal bovine serum (FBS) and incubated for 10 min at room temperature. Spleen lymphocytes were isolated from BALB/c mice 9 days after the booster vaccination and plated at 5 ×10^5^ cells/well (29). Lymphocytes were stimulated with 1 μg of Recombinant Delta/SARS-CoV-2 Spike Trimer protein (ACROBiosystems, SPN-C52He) or recombinant B.1.1.529/Omicron SARS-CoV-2 Spike Trimer protein (ACROBiosystems, SPN-C52Hz)and cultured for 20 h (37°C, 5% CO_2_). The plates were washed six times with wash buffer and incubated for 1 h with biotinylated anti-mouse IL-4/IL-5/IL-6/TNF-α/IL-2/IFN-γ antibody. The plates were washed six times and incubated for 1 h with Streptavidin-HRP. The final wash was followed by the addition of AEC substrate solution for 10 min. The chromagen was discarded and the plates were washed with water and dried in a dim place. Spot numbers were evaluated using ELIspotreader.

### 2.8 Lymphocyte population

RBMRNA-405-induced effects on proliferation and dynamics of immune cell populations were evaluated using Accuri C6 (BD Biosciences). For T-cell subset analysis, 1×10^6^ mice splenocytes (100 μl) were seeded per well into 96-well plates. Following two washes with PBS, the viability of cells was determined using Zombie Green™ Fixable Viability Kits (BioLegend, 423111). After washing once with FC buffer (PBS + 2% FBS), cells were resuspended in FC buffer. Then, splenocytes were stained with PerCP/Cyanine5.5 anti-mouse CD3 (BioLegend, 100218), PE anti-mouse CD8a (BioLegend, 100708), APC anti-mouse CD4 (BioLegend, 100412), PE anti-mouse CD62L (BioLegend, 161203), and APC anti-mouse/human CD44 (BioLegend, 103011). For GC response analysis, inguinal and iliac lymph nodes (LNs) were pooled and lymphocytes were isolated. Cells were treated with the same method as splenocytes except for the following antibodies: APC anti-mouse CD4 (BioLegend, 100412), PE anti-mouse CD185 (CXCR5) (BioLegend, 145503), PerCP/Cyanine5.5 anti-mouse CD278 (ICOS) (BioLegend, 117424), APC anti-mouse CD19 (BioLegend, 115512), PE anti-mouse CD95 (Fas) (BioLegend, 152608), PE anti-mouse CD138 (Syndecan-1) (BioLegend, 142504), APC anti-mouse/human CD45R/B220 (BioLegend, 103212), and PE anti-mouse CD79b (Igβ) (BioLegend, 132804).

### 2.9 Pseudovirus neutralization assay

The gene sequence of wild-type SARS-CoV-2 S protein or SARS-CoV-2 variant S protein is listed in **Table S3** and codon optimized for expression in human cells. SARS-CoV-2 pseudovirus neutralization assay was performed using an HIV-1 lentiviral packaging system following a similar approach that was previously reported (30). Plasmids expressing S protein and plasmids encoding luciferase-expressing lentivirus (pLV-Luc, pH1) were co-transfected into 293T cells using Lipofectamine 3000 Transfection Reagent (Thermo, L3000015). Cell suspensions enriched with the pseudotype virus were harvested after 48 h and TCID_50_ was measured. Pseudoviruses were stored at −80°C.

Serial diluted inactive serum was incubated with 300–600 TCID_50_ of the pseudovirus for 1 h at 37°C in 96-well plates. DMEM was used as cell control (CC) and lentiviral SARS-CoV-2 pseudoviruses were used as virus control (VC). After incubation, 4×10^4^ 293T-ACE2-p2A-mTagBFP2 cells (a cell line that stably expresses human ACE-2) were added and incubated for 48 h. Following this, cells were lysed, and luciferase signal was measured using the Luciferase assay system (Promega, PR-E4550) through a GloMax® 96 Microplate Luminometer (Promega). The neutralization potency (or inhibition rate) was calculated as follows: Inhibition ratio (%) = [1 – (mean RFU of Sample wells – mean RFU of CC wells)/(mean RFU of VC wells – mean RFU of CC wells)] × 100%; the neutralizing titers (pVNT titers) were calculated by the method of Reed–Muench (31).

### 2.10 Live virus neutralization assay

African green monkey kidney epithelial (Vero E6) cells (CRL-1587) were purchased from ATCC, which were cultured in Dulbecco’ s modified Eagle’s medium (DMEM, Gibco) with 10% fetal bovine serum (FBS), 100 U/ml penicillin, and 100 μg/ml streptomycin. The Omicron coronavirus (B.1.1.529 strain, TCID_50 = _10^-5.5^/100 μl) was clinically isolated from Guangzhou City Eighth People’s Hospital. Serum samples collected from immunized mice were inactivated at 56°C for 30 min and serially diluted with DMEM medium (GIBCO) in twofold steps. The diluted sera were mixed with 100 TCID_50_ B.1.1.529 strain in 96-well plates at a ratio of 1:1 (vol/vol) and incubated at 37°C for 1 h. Then, virus/serum mixtures were added to Vero-E6 cell monolayers in quadruplicate in 96-well plates and the plates were incubated for 3–5 days at 37°C in a 5% CO_2_ incubator. Cytopathic effect (CPE) of each well was recorded under microscopes, and the 50% neutralization Ab (NAb) titers were calculated according to the method of Reed and Muench (31).

### 2.11 SARS-CoV-2 challenge experiment

For the Delta or Omicron variant challenge, the Delta coronavirus (B.1.617.2 strain, TCID_50 = _10^–6.5^/100 μl) and the Omicron coronavirus (B.1.1.529 strain, TCID_50 = _10^–5.5^/100 μl) were clinically isolated from Guangzhou City Eighth People’s Hospital. K18-hACE2 mice (6–9 weeks) were divided into groups and injected intramuscularly with low-dose (5 μg/dose) vaccine, high-dose (20 μg/dose) vaccine, or physiological saline, respectively. All grouped animals were immunized twice on Day 0 and Day 21 before challenged intranasally with 10^3^ PFU of the SARS-CoV-2 Delta variant or 10^4^ PFU of the Omicron variant. The saline control group received the same SARS-CoV-2 challenge and mock-challenged group received cell culture medium. Mice were euthanized and the left lung and spleen tissues were collected at 5 or 7 dpi for histopathological examination, while the right lungs were collected to determine the viral titer using TCID_50_ assay according to the method of Reed and Muench (31).

### 2.12 Histopathology assay

For histopathology, left lung and spleen tissues from all animals were fixed in 4% neutral-buffered formaldehyde for 48 h, embedded in paraffin, sectioned, and stained with hematoxylin and eosin (H&E). Images were captured using Leica DM3000. Original magnification was 50× or 100×.

### 2.13 TCID_50_ assay

SARS-CoV-2 virus titers in lung homogenates were determined by TCID_50_ assay. Vero E6 cells were seeded in 96-well plates in serum-free media and incubated at 37°C with 5% CO_2_. Lung homogenates (four to eight replicates) were serially diluted in serum-free media and added to plates that were 95% confluent after a single wash. Cytopathic effects (CPEs) of each well were determined after 3–5 days. The TCID_50_/ml was calculated using the lowest dilution at which CPE was observed. Lung homogenate results were reported as TCID_50_/lobe.

### 2.14 Statistical analysis

Statistical analysis was performed with unpaired *t*-tests using GraphPad Prism 6.0 software. *p < 0.05; **p < 0.01; ***p < 0.001; ****p < 0.0001. Non-significant comparisons are not shown, unless otherwise noted as ns, not significant.

## 3 Results

### 3.1 RBMRNA-405 immunization elicits robust S-specific T-cell and germinal center (GC) B-cell responses

**Figure 1:**
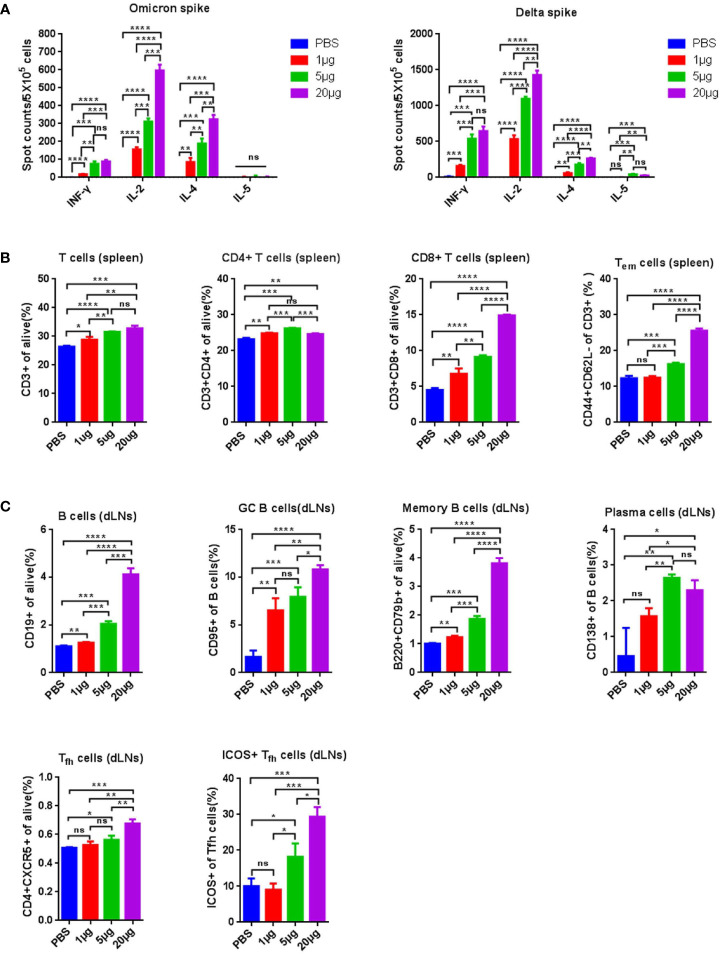
RBMRNA-405 elicited T-cell responses in mice. Groups of BALB/c mice (*n* = 3) were vaccinated intramuscularly (I.M.) with two doses of RBMRNA-405 (1, 5, or 20 μg) or PBS buffer at an interval of 21 days. **(A)** Total splenocytes were obtained 9 days post boost and were *ex vivo* re-stimulated with Omicron spike (left) or Delta spike (right), and splenocytes secreting IFN-γ, IL-2, IL-4, and IL-5 were measured by ELIspot assay. **(B)** Total splenocytes were obtained 9 days post boost and the percentages of lymphocyte subsets (CD3^+^, CD4^+^, CD8^+^, and Tem) were measured by flow cytometry. The values are means ± SD (*p*-values were determined by an unpaired two-tailed *t*-test, ns, not significant, *p < 0.05, **p < 0.01, ***p < 0.001, ****p < 0.0001. **(C)** Lymphocytes from draining lymph nodes (dLNs) were obtained 7 days post boost and the percentages of B cells (CD19+), GC B cells (CD19+/CD95+), MBCs (B220+/CD79b+), plasma cells (CD19+/CD138+), Tfh cells (CD4+/CXCR5+), and Tfh cells (CD4+/CXCR5+) among Tfh cells were determined by flow cytometry (*p*-values were determined by an unpaired two-tailed *t*-test, ns, not significant, *p < 0.05, **p < 0.01, ***p < 0.001, ****p < 0.0001.

To assess the induction of T- and B-cell immune responses by RBMRNA-405, BALB/c mice were intramuscularly vaccinated with two doses of RBMRNA-405 (1, 5, and 20 μg, respectively) 3 weeks apart. Lymphocytes from the spleen and draining lymph nodes (dLNs) were obtained 7–9 days post-boost to evaluate the T- and B-cell responses, particularly CD4^+^ T_fh_ cells that are crucial for the production of GCs that contribute to antigen-specific B-cell differentiation and long-lived PC responses (32, 33). Firstly, the T-cell cytokine profile of S-reactive cells were evaluated by ELIspot. Nine days following boost, we found that a T help (Th) 1-type response was elicited at all dose levels after stimulation with SARS-CoV-2 Delta spike demonstrated by increased levels of IL-2- and IFN-γ-producing cells (**Figure 1B**). Similarly, Omicron spike-stimulated splenocytes secreted significantly enhanced levels of IL-2, but low levels of IFN-γ in addition to the Th2-type cytokines IL-5 (**Figure 1A**). Although the IFN-γ secretion level induced by Omicron spike was lower, Th1-type cytokines induced by Omicron spike was generally higher than Th2-type cytokine levels. Of note, we found that Delta spike induced antigen-specific T-cell immune response was stronger than that of Omicron spike, which aligned with the previous reported results that also induced weaker cellular immunity (34). We hypothesize that such difference is a result of the different T-cell epitopes in the Omicron variant spike. To better capture the effects of RBMRNA-405 vaccination on the proliferation of T-cell populations, we developed a flow cytometry panel that showed increased frequencies of CD3^+^, CD4^+^, CD8^+^, and effector memory T cells (Tem) in all dose groups following immunization of RBMRNA-405 compared with the phosphate-buffered saline (PBS) control group (**Figures 1B**, **S1**). In particular, the 5- and 20-μg dose groups triggered a more significant increase in spleen CD8^+^ and Tem cells (*p* < 0.001, 5 μg; *p* < 0.0001, 20 μg) (**Figure 1B**). These data support the idea that RBMRNA-405 encoding both Delta-derived and Omicron-derived S protein activates Th1-biased T-cell immune response to guide humoral and cellular immunity.

We further evaluated the GC responses. B cells, GC B cells, MBCs, and PCs in draining lymph nodes (dLNs) following RBMRNA-405 immunization (**Figure 1C**), which were evaluated 7 days after the boost. Higher percentages of B cells (*p* = 0.0042, 1 μg; *p* = 0.0001, 5 μg; *p* < 0.0001, 20 μg), GC B cells (*p* = 0.0041, 1 μg; *p* = 0.0009, 5 μg; *p* < 0.0001, 20 μg), and MBCs (*p* = 0.0026, 1 μg; *p* = 0.0002, 5 μg; *p* < 0.0001, 20 μg) (**Figures 1C**, **S2**) were observed in dLNs from RBMRNA-405 vaccinated mice in all doses compared with the PBS group. Furthermore, the percentages increased in a dose-dependent manner. PCs also increased at all dose levels in the vaccinated group in comparison to the PBS control group (*p* = 0.0784, 1 μg; *p* = 0.0090, 5 μg; *p* = 0.0190, 20 μg) (**Figure 1C**). The generation of circulating MBCs and PCs can mount robust long-term immunity after antigen re-exposure through persistent production of antigen-specific NAbs. We also determined the percentage of T_fh_ cells and inducible costimulator (ICOS^+^) subsets induced by RBMRNA-405. T_fh_ cells regulate the proliferation, survival, and differentiation of GC B cells through the delivery of costimulatory molecules and cytokines, which impacts germinal center formation and facilitates generation of protective humoral immunity (33, 35). As shown in **Figures 1C**, **S3**, 7 days after receiving two doses of RBMRNA-405, T_fh_ cells and ICOS^+^ T_fh_ cells increased dose-dependently in dLNs. The increased frequencies of B cells, GC B cells, MBCs, PCs, and Tfh cells demonstrated RBMRNA-405-induced GC reactions, which has been also observed in previous mRNA vaccines (36, 37).

### 3.2 RBMRNA-405 immunization elicits S-directed antibodies with favorable IgG2a/IgG1 ratio against SARS-CoV-2 variants in mice

**Figure 2:**
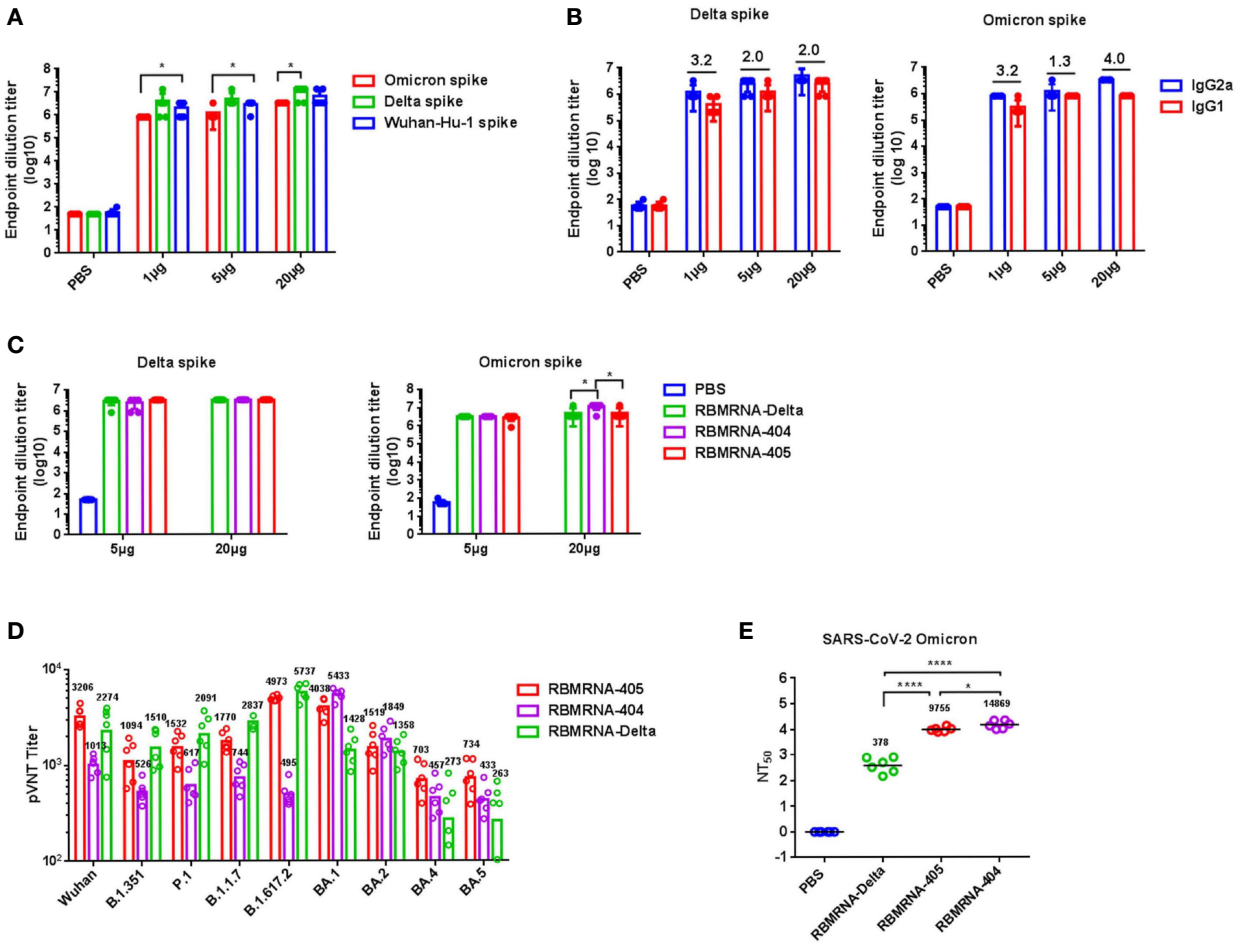
Compare the antibody responses in BALB/c mice after immunization with RBMRNA-404, RBMRNA-405, or RBMRNA-Delta. **(A, B)** Groups of BALB/c mice (*n* = 6) were intramuscularly vaccinated twice with 1-, 5-, or 20-μg doses of RBMRNA-405 or PBS buffer, respectively, over a 3-week interval. Sera were collected 2 weeks post-boost and humoral immune responses were assessed. IgG antibodies binding to Omicron, Delta, or Wuhan-Hu-1 spike proteins were measured by ELISA **(A)**. The values are means ± SD. Non-significant comparisons are not shown (*p*-values were determined by an unpaired *t*-test, *p < 0.05). Anti-Omicron or Delta spike antibodies of the IgG1 and IgG2a isotypes were assayed by IgG subclass ELISA, and IgG2a/IgG1 ratio values were calculated and indicated above the columns **(B)**. **(C)** Groups of BALB/c mice (*n* = 6) were intramuscularly vaccinated twice with 5- or 20-μg doses of RBMRNA-405, RBMRNA-404, RBMRNA-Delta, or PBS buffer, respectively, over a 3-week interval. Sera were collected 2 weeks post-boost, and IgG antibodies binding to Omicron and Delta spike proteins were measured by ELISA. The values are means ± SD. Non-significant comparisons are not shown (*p*-values were determined by an unpaired *t*-test, *p < 0.05). **(D)** Sera were collected from mice vaccinated with 20-μg vaccines, and pseudovirus cross-neutralization assays with Wuhan SARS-CoV-2, Alpha, Beta, Gamma, Delta, BA.1, BA.2, BA.4, and BA.5 pseudovirus were performed. Neutralizing antibody (NAb) titers calculated by the Reed–Muench method. The values are geometric mean. **(E)** Sera were collected from mice vaccinated with 20-μg vaccines, and live virus neutralization titers were measured using live SARS-CoV-2 (Omicron, B.1.1.529) plaque reduction neutralization assay. The values are geometric mean (*p*-values were determined by an unpaired *t*-test, *p < 0.05, ****p < 0.0001.

BALB/c mice were intramuscularly immunized twice over 21 days with 1-, 5-, or 20-μg doses of RBMRNA-405 or PBS buffer. Two weeks following the second dose, serum samples were collected to quantify binding immunoglobulin G (IgG) responses against Wuhan-Hu-1 (WA1/2020, WT), Omicron, and Delta spike proteins by ELISA (**Figure 2A**). Binding antibody was observed at all groups except the control group. IgG titers in serum against the Omicron spike reached five to six logs; those against the Delta spike reached above seven logs; and those against WA1/2020 spike ranged from six to seven logs. WA1/2020 and Delta S-binding IgG antibody level detected after RBMRNA-405 immunization were nearly equivalent (*p* > 0.05). However, RBMRNA-405 immunized sera showed relatively lowered cross-reaction with Omicron spike (*p* < 0.05). We also measured the IgG1 and IgG2a titers in the sera. RBMRNA-405 elicited high titers of Omicron or Delta S-specific IgG2a and IgG1. In agreement with the Th1/Th2 T-cell polarization, RBMRNA-405 elicited more Omicron or Delta S-binding IgG2a than IgG1 in all vaccinated groups, and the IgG2a/IgG1 ratios were calculated (**Figure 2B**). To better characterize the cross-neutralization capacity induced by RBMRNA-405 immunization, we also prepared a monovalent Delta-specific and Omicron-specific LNP-encapsulated mRNA vaccine (termed RBMRNA-Delta and RBMRNA-404, respectively) and compared the three vaccines’ ability to generate S-binding antibody and NAb responses. As shown in **Figure 2C**, at the dose of 5 μg, all three vaccines elicited equal cross-binding activities against Delta and Omicron spike. The 20-μg dose of RBMRNA-404 elicited slightly higher IgG titers in serum against the Omicron spike than RBMRNA-Delta and RBMRNA-405 immunizations of the same dose (*p* < 0.05). For NAb responses, SARS-CoV-2 pseudovirus cross-neutralization assays were employed. Serum collected from BALB/c mice immunized with RBMRNA-404 (20 μg) showed the most robust neutralizing potency against BA.1 Omicron pseudoviruses (GMT 5433) and relatively great neutralizing capacity against BA.2 Omicron (GMT 1849). However, only limited inhibition of WA1/2020 (WT) (GMT 1013) and other SARS-CoV-2 variants (GMTs were less than 1,000) was shown, which is consistent with the previous results of Omicron-specific mRNA vaccines that induced variant-specific NAbs in mice (38–40). In contrast, the bivalent vaccine against both Omicron and Delta, RBMRNA-405 (20 μg), induced higher titers of NAbs against WA1/2020 (WT) (GMT 3206), Beta (GMT 1094), Gamma (GMT 1532), Alpha (GMT 1770), Delta (GMT 4973), BA.4 (GMT 703), and BA.5 (GMT 734) variants than the monovalent Omicron-specific vaccine, RBMRNA-404. Serum collected from mice immunized with RBMRNA-Delta (20 μg) showed the most robust neutralizing potency against Delta pseudoviruses (GMT 5737). Surprisingly, higher neutralizing activity against Beta (GMT 1510), Gamma (GMT 2091), and Alpha (GMT 2837) variants was shown as compared to RBMRNA-405, but reduced neutralization against BA.1, BA.4, and BA.5 variants (**Figure 2D**). Although the capacity to neutralize the other variants was decreased, RBMRNA-405 generated antibody with broadly neutralizing activity.

To further compare the neutralizing activities of the three vaccines, RBMRNA-404, RBMRNA-405, and RBMRNA-Delta, live virus neutralization assay was employed (**Figure 2E**). At the 20-μg dose, RBMRNA-404 and RBMRNA-405 vaccination showed high titers of NAbs against the Omicron variant with GMTs of 14,869 and 9,755, respectively. The RBMRNA-404 vaccine group showed higher titers of NAbs compared with the RBMRNA-405 vaccine group (*p* = 0.0314), which is probably due to the half dose of Omicron mRNA in the RBMRNA-405 vaccine. As expected, RBMRNA-Delta vaccination induced low serum NAbs against Omicron strains, with the GMT of 378 (**Figure 2E**). Taken together, the bivalent mRNA vaccine candidate, RBMRNA-405, generated broader NAb responses against Delta, Omicron, and other variants, compared to the variant-specific mRNA vaccines.

### 3.3 RBMRNA-405 immunization boosts immune responses against the Omicron variant in WA/1/2020 inactivated vaccinated mice

**Figure 3:**
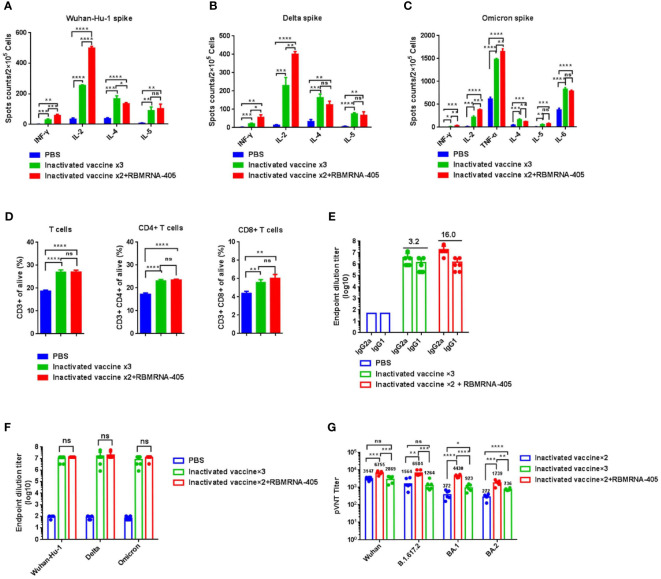
RBMRNA-405 boosts immune responses for SARS-CoV-2 Omicron variant. BALB/c mice were I.M. vaccinated with two doses of 3 μg of inactivated whole-virion SARS-CoV-2 vaccine (CoronaVac, Sinovac) or PBS buffer at week 0 and week 3, and 24 weeks after the second vaccine dose, mice were boosted with 3-μg doses of inactivated vaccine or 20-μg doses of RBMRNA-405. T- cell response and antibody response were compared between groups. **(A–C)** Seven days following vaccination with a third dose, splenocytes were re-stimulated with Wuhan-Hu-1 spike **(A)**, Delta spike **(B)**, or Omicron spike **(C)**, and splenocytes secreting IFN-γ, IL-2, IL-4, and IL-5 were measured by ELIspot assay (*n* = 3). **(D)** The percentages of lymphocyte subsets (CD3^+^, CD4^+^, and CD8^+^) were measured by flow cytometry (*n* = 3). **(E)** Sera were collected 2 weeks after the third dose, anti-Omicron spike antibodies of the IgG1 and IgG2a isotypes were assayed by IgG subclass ELISA, and IgG2a/IgG1 ratio values were calculated and indicated above the columns (*n* = 6). **(F)** Sera were collected 2 weeks after the third dose and IgG antibodies binding to Omicron, Delta, or Wuhan-Hu-1 spike were measured by ELISA (*n* = 6). The values are means ± SD (*p*-values were determined by an unpaired two-tailed *t*-test, ns, *p* > 0.05). **(G)** Sera were collected 24 weeks after the second dose and 2 weeks after the third dose, respectively; pseudovirus cross-neutralization assays with Wuhan SARS-CoV-2, Delta, BA.1, and BA.2 variants were performed (*n* = 6). Neutralizing antibody (NAb) titers are calculated by the Reed–Muench method. The values are geometric mean (*p*-values were determined by an unpaired two-tailed *t*-test. *p < 0.05, **p < 0.01, ***p < 0.001, ****p < 0.0001).

As a large number of people have received two doses of the inactivated whole-virion SARS-CoV-2 vaccine, we further assessed whether a third dose of RBMRNA-405 could boost immune responses against Omicron variants. Groups of BALB/c mice were vaccinated intramuscularly twice with 3-μg doses of inactivated whole-virion SARS-CoV-2 vaccine (CoronaVac, Sinovac) at Week 0 and Week 3, and 24 weeks after the second dose, mice were boosted with 3-μg doses of inactivated vaccine or 20-μg doses of RBMRNA-405. Seven days following immunization with a third dose, lymphocytes from the spleen were obtained and T-cell responses were compared between groups. As shown in **Figures 3A–C**, regardless of restimulation with Wuhan-Hu-1 (WA1/2020, WT), Delta, or Omicron spike, the levels of IFN-γ- and IL-2-producing cells in the RBMRNA-405 booster group were higher than in the inactivated vaccine booster group, whereas the levels of IL-4-producing cells in the inactivated vaccine booster group were higher than the RBMRNA-405 booster group. Although there were no significant differences between the groups in IL-5 secretion, these data indicate that RBMRNA-405 as a booster induced a Th1-type biased T-cell response. Additionally, flow cytometry results showed an increase in CD3^+^, CD4^+^, and CD8^+^ T cells in all immunized groups compared with the PBS control group with no measurable difference between the inactivated vaccine booster group and the RBMRNA-405 booster group (**Figure 3D**). Fourteen days following immunization with a third dose, serum was collected to measure the IgG1 and IgG2a titers in sera (**Figure 3E**). The IgG2a/IgG1 ratios were higher in the RBMRNA-405 booster group (16.0) than in the inactivated vaccine booster group (3.2), which is consistent with the Th1/Th2 T-cell polarization (**Figures 3A–C**). As a booster, RBMRNA-405 and inactivated vaccine induced nearly equivalent IgG responses against WA1/2020, Delta, and Omicron spike proteins evaluated by ELISA as shown in **Figure 3F** (*p* > 0.05). We then assessed the NAb titers pre- and post-boost by pseudovirus neutralization assays (**Figure 3G**). Heterologous prime-boost regimens with RBMRNA-405 induced NAb GMTs of 6,755, 6,584, 4,430, and 1,739 against WA1/2020 (WT), Delta, BA.1, and BA.2 Omicron, respectively, which were 2.1-, 4.2-, 11.9-, and 6.4-fold higher than pre-boost groups. In comparison, the GMTs against WA1/2020 (WT), Delta, BA.1, and BA.2 Omicron were 2,869, 1,264, 923, and 736 in the inactivated vaccine booster group. Our data suggest that RBMRNA-405 as a booster is more effective in eliciting virus neutralizing activity against VOCs, particularly Omicron.

### 3.4 Protective efficacy of RBMRNA-405 against SARS-CoV-2 variant challenge

**Figure 4:**
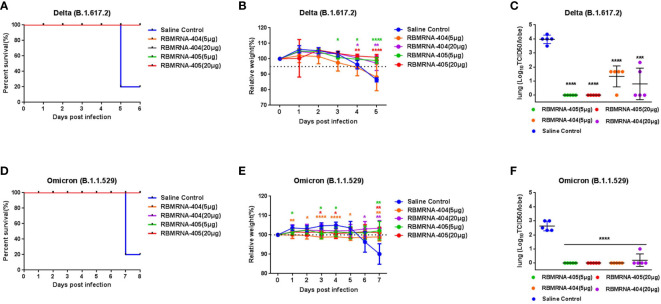
RBMRNA-405 reduced viral burden in the lungs in SARS-CoV-2 variant-challenged mice. Groups of K18-hACE2 mice (*n* = 5 per group) were intramuscularly vaccinated twice with 5- or 20-μg doses of RBMRNA-405, RBMRNA-404, or physiological saline on Day 0 and Day 21. Day 32 after dose 1, K18-hACE2 mice were challenged with 1×10^3^ plaque-forming units (PFU) of Delta (B.1.617.2) live virus, or on Day 52 after dose 1, another group of K18-hACE2 mice were challenged with 1×10^4^ PFU of Omicron (B.1.1.529) live virus. **(A, D)** Survivorship curves were shown. **(B, E)** Relative weight was measured as a percent of starting weight at the indicated days post-infection with SARS-CoV-2 variants. The values are means ± SD (*p*-values were determined by multiple *t*-test using GraphPad Prism 6.0 software, *p < 0.05, **p < 0.01, ***p < 0.001, ****p < 0.0001). Non-significant comparisons are not shown. **(C, F)** Five days after Delta challenge or 7 days after Omicron challenge, viral titers in right lungs were quantified by TCID_50_ assay. The values are means ± SD (*p*-values were determined by an unpaired *t*-test, *p < 0.05, **p < 0.01, ***p < 0.001, ****p < 0.0001).

**Figure 5:**
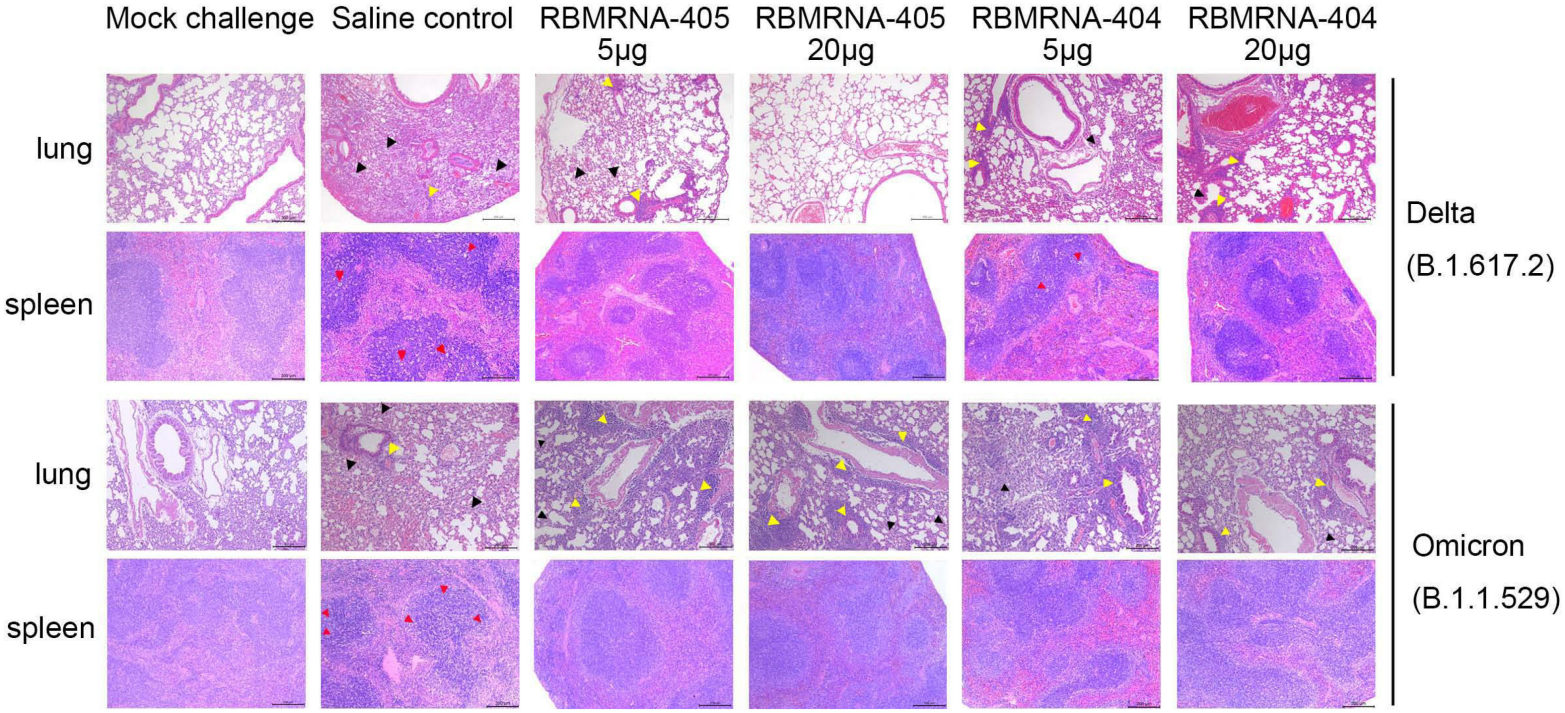
RBMRNA-405 reduced lung and spleen pathology in K18-hACE2 transgenic mice. Groups of K18-hACE2 mice (*n* = 5 per group) were intramuscularly vaccinated twice with 5- or 20-μg doses of RBMRNA-405, RBMRNA-404, or physiological saline on Day 0 and Day 21. On Day 32 after dose 1, K18-hACE2 mice were challenged with 1×10^3^ PFU of Delta (B.1.617.2) live virus, and on Day 52 after dose 1, K18-hACE2 mice were challenged with 1×10^4^ PFU of Omicron (B.1.1.529) live virus. The saline control group received the same SARS-CoV-2 challenge and additional mice were mock-challenged with cell culture medium. Five or 7 days after SARS-CoV-2 challenge, left lungs and spleens were harvested for hematoxylin and eosin (H&E) staining and examination of histopathologic changes. Representative images of each group are shown. The black arrows indicate the lesions of infected animals, the yellow arrows indicate the inflammatory cell infiltration, and the red arrows indicate the apoptosis cells (Mock challenge and Saline control, original magnification 100×; RBMRNA-404 and RBMRNA-405, magnification 50×; scale bar: 200 μm).

To compare the protective effect of RBMRNA-405 with monovalent RBMRNA-404, groups of 6- to 9-week-old K18-hACE2 transgenic mice (*n* = 5 per group) (41) were immunized with 5 or 20 μg of RBMRNA-405 or RBMRNA-404 *via* intramuscular injections and boosted with identical immunizations 3 weeks later. The non-immunized control group was injected with physiological saline. Animals were challenged with 1×10^3^ plaque-forming units (PFU) of Delta live virus *via* the intranasal route 32 days after the first dose or challenged 52 days after the first dose with 1×10^4^ PFU of Omicron live virus by the intranasal route. Clinical signs were recorded during the observation after the SARS-CoV-2 variant challenge. At 5 or 7 days post-infection (dpi), mice in each group were euthanized for lung viral titer measurements (**Figure 4**) and lung and spleen tissue histopathology analysis (**Figure 5**). For the Delta variant challenge, four of five mice in the saline control group died within 5 days, whereas all mice immunized with RBMRNA-405 or RBMRNA-404 survived, indicating fully protected transgenic mice from SARS-CoV-2 lethality (**Figure 4A**). Animals who received physiological saline started losing weight at 4 dpi. In comparison, 5 or 20 μg of RBMRNA-405 and 20 μg of RBMRNA-404 prevented weight loss during a 5-day observation. However, 5 μg of RBMRNA-404 failed to prevent weight loss at 5 dpi (**Figure 4B**). After Delta infection, mice in the saline control group showed high infectious viral titers (3.967Log10 TCID_50_/lobe) in the right lungs, whereas we were unable to detect infectious virus in lung tissue from RBMRNA-405 vaccinated animals (**Figure 4C**). Notably, infectious virus could be isolated in RBMRNA-404 vaccinated mice at 5 dpi, although RBMRNA-404 vaccine significantly reduced titers of infectious virus in lung tissue (*p* < 0.0001, 5 μg; *p* = 0.0003, 20 μg). For the Omicron variant challenge, 100% survival was observed 7 dpi for the groups immunized with RBMRNA-405 or RBMRNA-404 (**Figure 4D**). Mice in the control group lost approximately 10% of initial weight at 7 dpi; in contrast, slow increases of body weight were observed in RBMRNA-405-immunized mice, while mice immunized with 5 μg of RBMRNA-404 maintained their body weight (**Figure 4E**). S-binding IgG was consistently detected on Day 14, Day 21, Day 28, Day 32, Day 52, and Day 66 post-primary vaccination. Immunization by either dose of RBMRNA-405 or RBMRNA-404 resulted in high IgG titers and persisted to Day 66 (**Tables S1**, **S2**). We also quantified the viral titers in the lungs of mice challenged with Omicron variant (**Figure 4F**). Similar to the Delta variant challenge, the amount of infectious virus was undetectable in lungs in RBMRNA-405 vaccinated animals. Mice immunized with 20 μg or 5 μg of RBMRNA-404 also showed elimination of the infectious viruses in lungs, indicating better protection from Omicron infection than Delta. Only one mouse in the high-dose group showed viral titers (1.0Log_10_ TCID_50_), which was probably due to the individual differences. Cumulatively, these data demonstrate that immunization with 5 μg or 20 μg of RBMRNA-405 produced 100% protection against death and could efficiently block the infectious virus of Delta and Omicron variants in mice. RBMRNA-404 vaccination only partially blocked the infectious virus of the Delta variant.

We examined lung and spleen tissues histologically on day 5 or 7 post-challenge. Lung sections obtained from control animals challenged with Delta or Omicron showed moderate-to-severe interstitial pneumonia, characterized by lung interval broadened, lung interval fractured with large amounts of macrophage and lymphocyte infiltration, visible protein-rich edema in alveolar cavity, and perivascular leukocyte infiltration. In contrast, for mice challenged with the Delta variant, RBMRNA-405-immunized mice presented only focal and minor histopathological changes in a dose-dependent manner. Mice immunized with two 5-μg doses of RBMRNA-405 showed lung interval slightly broadened, moderate or mild perivascular leukocyte infiltration, without visible alveolar serous fluid exudates. For the 20-μg dose, the majority of mice (three of five) did not develop lung pathology after the Delta challenge, with histological findings similar to mock-challenged mice. Moreover, control animals showed a profound apoptosis of the lymphocytes in the white pulp of the spleen, whereas no lesions or pathology was observed in spleen tissue of RBMRNA-405 vaccinated animals. As a comparison, mice vaccinated with 5 or 20 μg of RBMRNA-404 showed mild interstitial pneumonia with protein transudates and moderate or mild perivascular leukocyte infiltration. For the pathology of spleen, mice vaccinated with 5 μg of RBMRNA-404 showed a mild apoptosis of the lymphocytes in the spleen and mice vaccinated with 20 μg of RBMRNA-404 showed normal spleen. In line with the viral titers, both RBMRNA-405 and RBMRNA-404 immunized mice showed alleviative lung and spleen injury, and RBMRNA-405 showed better protection from Delta variant infection-induced severe disease than RBMRNA-404.

Likewise, for mice challenged with the Omicron variant, RBMRNA-405 and RBMRNA-404 immunized mice showed mild-to-moderate interstitial pneumonia after Omicron infection. Of note, we also observed moderate-to-severe perivascular inflammatory infiltrates in the mice immunized with 5 or 20 μg of RBMRNA-404 and RBMRNA-405. Monocytes and lymphocytes were the main components of the cellular infiltrates around the blood vessels. Despite some differences in perivascular inflammatory infiltrates, we did not observe any overt lung pathology in the RBMRNA-404 or RBMRNA-405 immunized mice after the Omicron challenge, when compared to the saline control mice. Furthermore, a profound apoptosis of the lymphocytes was observed in the spleen in control mice, and no pathology was observed in the spleen of all vaccinated animals. Our results demonstrated the protective effects of RBMRNA-405 and RBMRNA-404 immunization in suppressing Omicron-induced pathological changes in the lungs, and there is no observed difference at the protection efficacy against Omicron variant between the two vaccines. Overall, the results showed that the bivalent RBMRNA-405 vaccine with better antigen coverage could efficiently block the infection of both variants of SARS-CoV2 in mice.

## 4 Discussion

The rapid spread of Omicron variant around the world and the diminished protection efficacy of pre-existing vaccines based on the ancestral strain of SARS-CoV-2 stress the importance and urgency of developing updated vaccines that are effective against emerging variants. Therefore, developing bivalent or multivalent SARS-CoV-2 vaccine with broad-spectrum efficacy is critical. In this study, we develop a bivalent LNP-encapsulated mRNA vaccine, RBMRNA-405, containing a 1:1 mix of pseudouridine nucleoside-modified mRNAs encoding the Omicron and Delta spike protein with 2P mutation. We evaluate the T-cell and B-cell responses, antibody responses, and protective efficacy against SARS-CoV-2 VOCs in transgenic mice, particularly the Omicron and Delta variants.

The safety profile of a candidate vaccine is a key component in its evaluation. As vaccine-associated enhanced disease (VAED) has a history in the development of formalin-inactivated respiratory syncytial virus (RSV) vaccines (42), it should be taken into account to assess the safety of candidate vaccines. Recent studies on SARS and SARS-CoV-2 showcased the importance of Th1-based immune response in providing the protection against infection and its critical role in reducing the production of VAED (43–46). In addition, Th2 response-related lung immunopathology has been reported for SARS-CoV, MERS-CoV, and RSV vaccines following viral infections (47–50). Such immune response by Th1/Th2-type cytokines and its isotypic bias can be measured by IgG1 and IgG2a. Here, we observed that RBMRNA-405 elicited higher levels of IgG2a than IgG1 subclass S-binding antibodies and increased secretion of IFN-γ and IL-2 by T cells in the splenocytes of immunized mice with minimal or no IL-4 and IL-5 observed across all three doses, indicating that RBMRNA-405 immunization induced Th1-type skewed immune response. Although we observed moderate perivascular inflammatory infiltrates in Omicron-specific monovalent vaccine RBMRNA-404- or bivalent vaccine RBMRNA-405-immunized mice following the Omicron variant challenge, RBMRNA-405 fully protected transgenic mice from SARS-CoV-2 lethality and severe disease. Accumulated data suggest that non-protective antibodies induce Th2 polarization with a deficit of cytotoxic T cells, which leads to VAED and is often accompanied by eosinophilic infiltration in the lungs (51). In our study, the infiltrates were monocytes and lymphocytes without eosinophil components in immunized challenged animals. As monocytes and macrophages are sentinel cells that sense invasive infection and release the potent inflammatory mediators (52), we speculate that viral replication likely occurred early after Omicron challenge, which led to the promotion of MCP1 and IL-8 production by the existing S-IgG as well as proinflammatory monocyte/macrophage recruitment and accumulation prior to viral clearance. Further studies are needed to elucidate the pathomechanism.

As vaccine-elicited CD4^+^ T cells recognize peptide epitopes distributed throughout the SARS-CoV-2 spike protein, they are less likely to be affected by antibody escape mutations in variant viral strains and therefore provide protection from severe disease even if the NAbs are no longer effective (53–55). The pivotal role of virus-specific memory T cells in providing broad and long-term protection against severe SARS-CoV was reported (56). Numerous studies previously demonstrated rapid and universal induction of antigen-specific T-cell responses by mRNA vaccination (55, 57–60). We observed a similar pattern with increased counts of activated CD4^+^ T cells, CD8^+^ T cells, and Tem cells in RBMRNA-405-immunized animals. This indicates that RBMRNA-405-induced T-cell responses may play an important role in protecting against viral reinfection and prevent severe disease during antibody escape. Given that RBMRNA-405 initiated strong Th1-type biased response in this study, we also assessed the germinal center responses and T_fh_ cell responses induced by RBMRNA-405 vaccination to fully characterize CD4^+^ T-cell differentiation (61). We immunized BALB/c mice with 1, 5, or 20 μg of RBMRNA-405 at Days 0 and 21 and measured percentage changes of B cells, GC B cells, MBCs, and plasma cells in dLNs 7 days post-boost by flow cytometry. Consistent with a previous study (37), immunized mice showed significant induction of increased GC B cells when compared to unimmunized animals. As expected, the percentages of MBCs and plasma cells were elevated, in which GC B cells can differentiate into MBCs or antibody-secreting plasma cells. The increased percentages of T_fh_ cells and ICOS^+^ T_fh_ cells in dLNs in immunized mice indicate the establishment of a T_fh_ program induced by RBMRNA-405. As the key regulator of GC responses, T_fh_ cells regulate proliferation, survival, and differentiation of GC B cells into MBCs and plasma cells through expressing costimulatory markers such as CD40L and ICOS and producing IL-2 (33, 62). Therefore, these results reveal that RBMRNA-405 immunization could foster potent antigen-specific T-cell and GC B-cell responses, which may mediate NAb generation and long-term adaptive immunity against Omicron and Delta variants.

There are several studies that evaluated the immunogenicity of RBD-based or spike-based mRNA vaccines specific to Omicron, which consistently found that Omicron-specific mRNA vaccines elicited a narrow neutralization spectrum against the Omicron variant (38–40). Previous recombinant protein vaccine based on Omicron S1 elicited reduced neutralizing activity, weaker T-cell response, GC-B cell response, and T_fh_ cell response than wild-type S1 recombinant protein vaccine (34), a likely result of the marked antigenic changes in Omicron spike, especially those in the RBD. The results indicate that Omicron spike protein alone may not be a suitable antigen for designing vaccines against SARS-CoV-2 variants, including Omicron. In our study, the bivalent mRNA vaccine, RBMRNA-405, containing halved dose of mRNA of each respective antigen, induced similarly high Nab against Omicron and Delta as full-dose Omicron- or Delta-specific vaccines. Moreover, RBMRNA-405 generated antibodies with broad neutralizing activity against all pseudovirus VOCs, demonstrating that bivalent vaccine with diversified antigen coverage, such as RBMRNA-405, may provide better broad-spectrum protection against emerging variants than monovalent vaccines. It is interesting to note that there is a lowered pseudovirus neutralizing ability against BA.2 than BA.1 across the three vaccine candidates. This could be a result of the different mutations among these two lineages as a previous study found that Omicron BA.2 has an absence of amino acid deletion 69–70 in the spike protein compared to BA.1, which has been associated with S gene target failure (63).

Additionally, we compared the Omicron-specific vaccine, RBMRNA-404, with RBMRNA-405 in the lethal challenge study, in which RBMRNA-404 completely protected immunized mice from the Omicron variant challenge, but could not eliminate infectious viruses in lungs after the Delta challenge. In comparison, RBMRNA-405 fully protected immunized mice from both the Delta and Omicron challenge. Both vaccines (5 or 20 μg) showed significantly reduced viral titers in the lungs of K18-ACE2 mice with only focal and mild histopathological changes following lethal challenges with 1×10^3^ PFU of SARS-CoV-2 Delta or 1×10^4^ PFU of Omicron, whereas virus replicated to reasonably high levels in the lungs of control animals 5 or 7 dpi and moderate-to-severe interstitial pneumonia was shown in the lung pathology of control animals. Interestingly, we found that infectious Omicron viral titer was lower (approximately 2.632Log TCID_50_/lobe) than Delta viral titer (approximately 3.967Log TCID_50_/lobe) in the lungs of control animals, though a higher dose (1×10^4^ PFU) of Omicron intranasal challenge was administered. This is possibly because Omicron is less pathogenic in mice and hamsters (64, 65).

We also tested RBMRNA-405 as a booster shot following two doses of inactivated whole-virion WA1/2020 SARS-CoV-2 vaccine against Omicron. Mice boosted with a third dose of RBMRNA-405 elicited higher levels of IFN-γ- and IL-2-secreting cells than the inactivated vaccine booster, regardless of the variant (WA1/2020, Delta, or Omicron spike) used for restimulation. IgG2a/IgG1 ratios were also higher in the RBMRNA-405 booster group, suggesting that RBMRNA-405 induced a Th1-type biased T-cell response. A third dose of RBMRNA-405 increased S-directed NAb GMTs against BA.1 and BA.2 Omicron by 11.9- and 6.4-fold higher than the pre-boost group, respectively, in pseudovirus neutralization assay while the homologous booster of inactivated vaccine improved 2.5- and 2.7-fold, respectively. This relatively low serum antibody titer against BA.1 and BA.2 in the homologous booster of inactivated vaccine is likely a result of NAb evasion manifested by the extensive number of mutations in Omicron. This result is consistent with literature that reported reduced virus neutralizing activity against BA.1 Omicron in individuals that received three doses of inactivated vaccine (66).

In summary, here we provide a detailed evaluation of the immunogenicity and protection efficacy of RBMRNA-405. Our data demonstrate that two doses of RBMRNA-405 elicit potent T- and B-cell immune responses to full-length S protein and broad-spectrum NAbs in immunized mice. RBMRNA-405 vaccination can provide protection from both Delta and Omicron variant challenge and support further development of this vaccine to test immunogenicity and efficacy in NHP and humans.

## 5 Limitations of the study

The broad-spectrum efficacy of the bivalent vaccine RBMRNA-405 was only evaluated by pseudovirus cross-neutralization assays due to the unavailability of SARS-CoV-2 in most VOC live virus in the BSL-3 laboratory. Follow-up experiments to assess NAb titers against other VOC live viruses are needed for stronger verification of antiviral activities of antibodies. We observed moderate perivascular inflammatory infiltrates in the RBMRNA-404 or RBMRNA-405 immunized mice after the Omicron challenge. We speculate that viral elimination could be associated with the proinflammatory macrophage decline and restoration of normal tissue architecture. However, we did not confirm the conclusion in our study due to limited experimental conditions. Further studies are needed to confirm the speculation and elucidate the pathomechanism. We challenged K18-hACE2 mice with Delta and Omicron BA.1, respectively. Currently, Omicron subvariants including BQ.1, BQ.1.1, BF.7, and XBB are emerging. Therefore, further infection experiments using these strains may be informative to understand the scope of protection against emerging VOCs.

## Data availability statement

The original contributions presented in the study are included in the article/**Supplementary Material**. Further inquiries can be directed to the corresponding authors.

## Ethics statement

BALB/c mice experiments were performed in strict accordance with the requirements of Guangdong experimental animal management regulations and Institutional Animal Care and Use Committee (IACUC Approval No.: IACUC-2021-026). K18-hACE2 transgenic mice were used and the experiments were performed in accord with protocols approved by The Animal Care and Use Committee of Guangzhou Medical University (Acceptance number: 2018-297).

## Author contributions

BZ and ZiY conceived the project and designed the experiments. QM, ML, LM, HZ, HLZ, YW, and ZeY conducted the experiments and analyzed the data. JW and WX supervised the production of GMP grade vaccines. QM, and JG conducted the K18-hACE2 mice challenge experiments and coordinated the challenge experiment in ABSL-3. WY and ZiY supervised the challenge experiment. ML and CZ wrote the original draft. CZ and BZ reviewed and revised the original draft. All authors contributed to the article and approved the submitted version.

## Supplementary material

Supplementary Figure 1 Representative staining of the memory T cell subsets, related to . **(A)** Gating strategy for the Tem cell (CD3+CD44+CD62L-) populations. **(B)** Representative flow cytometry plots showing Tem cells elicited by 1, 5 and 20 μg RBMRNA-405 immunization.

Supplementary Figure 2 Representative staining of lymph node GC B cell and Memory B cell population, related to ; **(A)** Gating strategy for lymph node Memory B cell populations. **(B)** Representative flow cytometry plots showing Memory B cells elicited by 1, 5 and 20 μg RBMRNA-405 immunization

Supplementary Figure 3 Representative staining of lymph node T_fh_ cell and ICOS+ T_fh_ cell population, related to **; (A)** Gating strategy for T_fh_ cell (CD4+ CXCR5+) and ICOS+ T_fh_ cell (CD4+ CXCR5+ ICOS+) populations. **(B** Representative flow cytometry plots showing T_fh_ cells and ICOS+ T_fh_ cells elicited by 1, 5 and 20 μg RBMRNA-405 immunization.

Supplementary Table 1 IgG titers against Wuhan SARS-CoV-2 spike protein following immunization with the RBMRNA-404. Three K18-hACE2 mice in each group were used for IgG titers detection. K18-hACE2 mice were intramuscularly vaccinated twice with 5 or 20 μg doses of RBMRNA-404 on Day 0 and Day 21. S-binding IgG was constantly detected on Day14, Day21, Day28, Day32, Day52 and Day66 post the primary vaccination.

Supplementary Table 2 IgG titers against Wuhan SARS-CoV-2 spike protein following immunization with the RBMRNA-405. Three K18-hACE2 mice in each group were used for IgG titers detection. K18-hACE2 mice were intramuscularly vaccinated twice with 5 or 20μg doses of RBMRNA-405 on Day 0 and Day 21. S-binding IgG was constantly detected on Day14, Day21, Day28, Day32, Day52 and Day66 post the primary vaccination.
