# Supplementary figures and images for: SARS-CoV-2 bivalent mRNA vaccine with broad protection against variants of concern
Source: Front Immunol. 2023 May 24;14:1195299. doi: 10.3389/fimmu.2023.1195299 (PMC10244545; doi:10.3389/fimmu.2023.1195299)

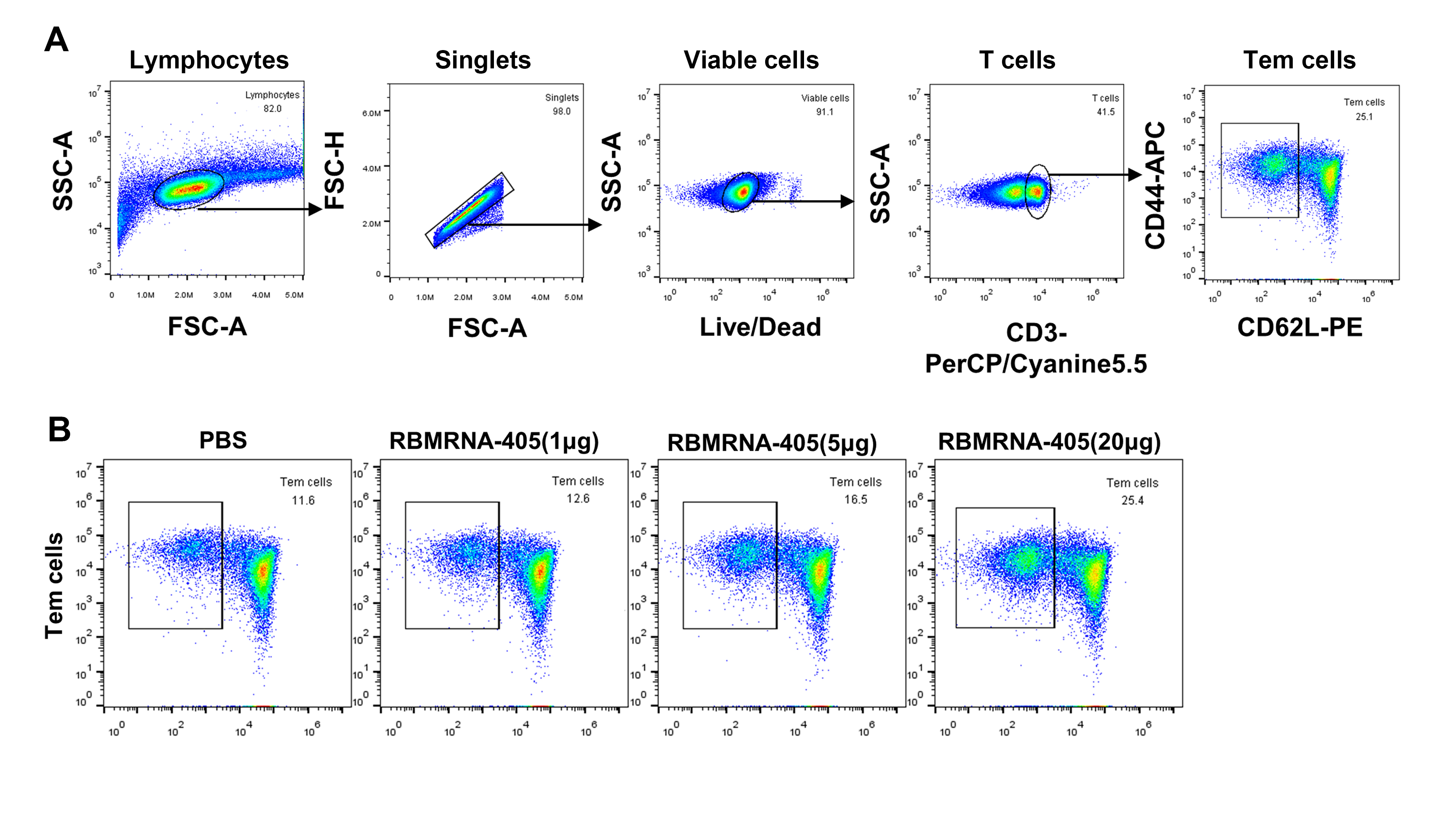

Supplement: Supplementary Figure 1 — Representative staining of the memory T cell subsets, related to . (A) Gating strategy for the Tem cell (CD3+CD44+CD62L-) populations. (B) Representative flow cytometry plots showing Tem cells elicited by 1, 5 and 20 μg RBMRNA-405 immunization. [file Image_1.jpeg]

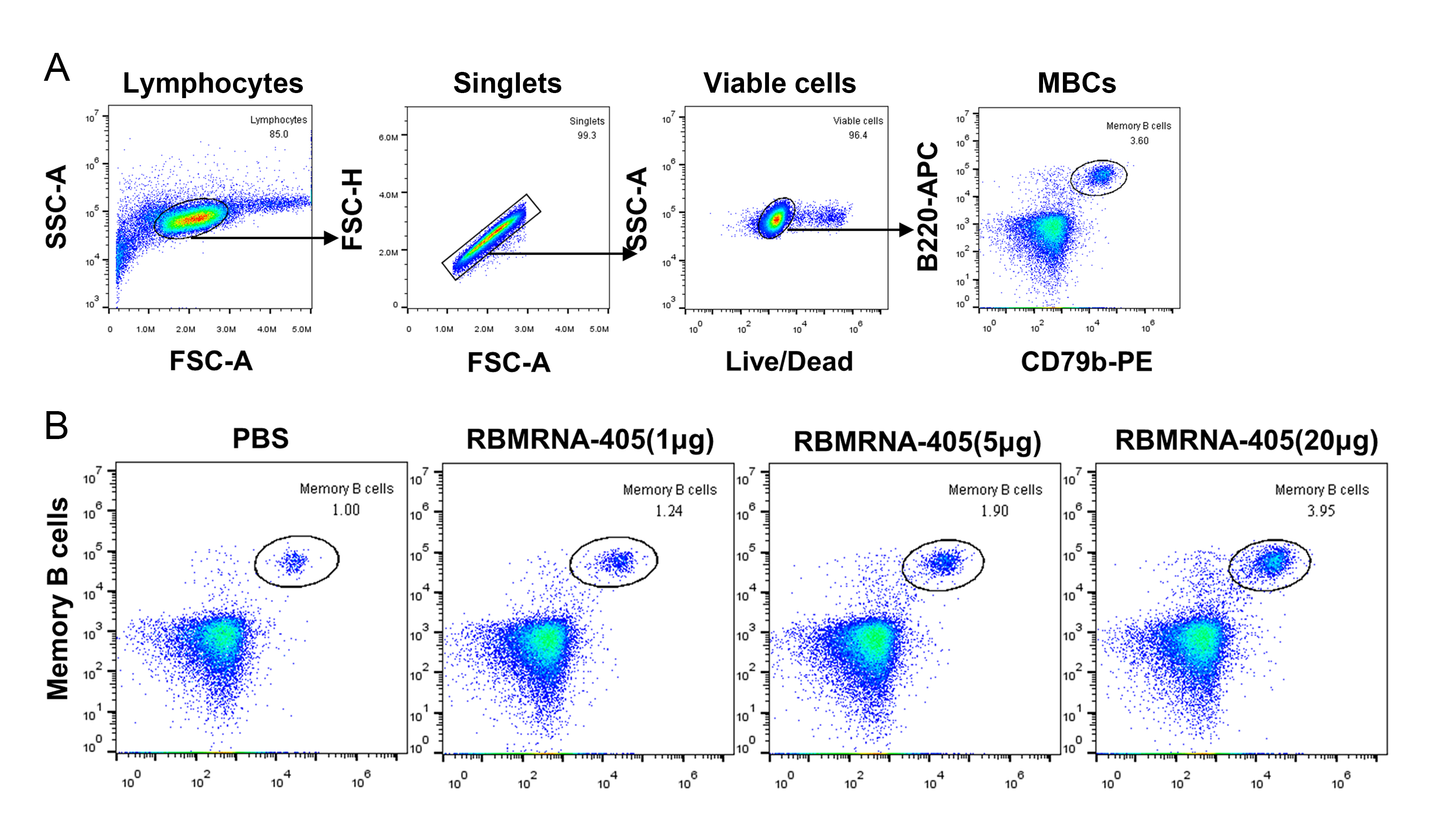

Supplement: Supplementary Figure 2 — Representative staining of lymph node GC B cell and Memory B cell population, related to ; (A) Gating strategy for lymph node Memory B cell populations. (B) Representative flow cytometry plots showing Memory B cells elicited by 1, 5 and 20 μg RBMRNA-405 immunization [file Image_2.jpeg]

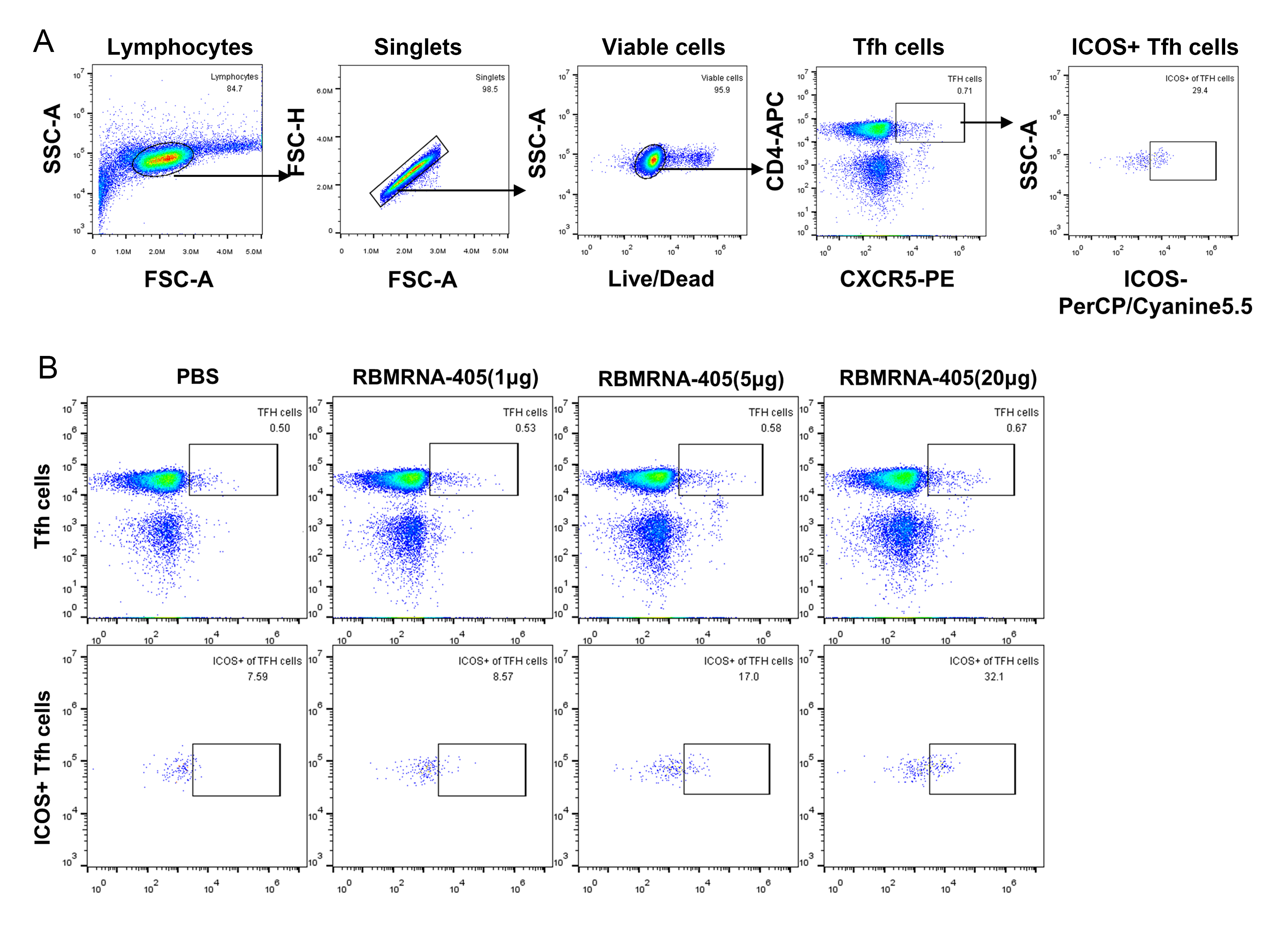

Supplement: Supplementary Figure 3 — Representative staining of lymph node Tfh cell and ICOS+ Tfh cell population, related to ; (A) Gating strategy for Tfh cell (CD4+ CXCR5+) and ICOS+ Tfh cell (CD4+ CXCR5+ ICOS+) populations. (B Representative flow cytometry plots showing Tfh cells and ICOS+ Tfh cells elicited by 1, 5 and 20 μg RBMRNA-405 immunization. [file Image_3.jpeg]
