# Supplementary material for: SARS-CoV-2 bivalent mRNA vaccine with broad protection against variants of concern
Source: Front Immunol. 2023 May 24;14:1195299. doi: 10.3389/fimmu.2023.1195299 (PMC10244545; doi:10.3389/fimmu.2023.1195299)
Supplement: Supplementary Table 1 — IgG titers against Wuhan SARS-CoV-2 spike protein following immunization with the RBMRNA-404. Three K18-hACE2 mice in each group were used for IgG titers detection. K18-hACE2 mice were intramuscularly vaccinated twice with 5 or 20 μg doses of RBMRNA-404 on Day 0 and Day 21. S-binding IgG was constantly detected on Day14, Day21, Day28, Day32, Day52 and Day66 post the primary vaccination. [file Table_1.docx]

**TableS1.** **IgG titers against Wuhan SARS-CoV-2 spike protein following immunization with the RBMRNA-404.**

| **Group** | **Sample** | **iD14** | **iD21** | **iD28** | **iD32** | **iD52** | **iD66** |
| --- | --- | --- | --- | --- | --- | --- | --- |
| **RBMRNA-404（5μg）** | C11 | 1.00E+04 | 1.00E+03 | 1.00E+06 | 1.00E+05 | 1.00E+05 | 1.00E+04 |
|  | C12 | 1.00E+03 | 1.00E+02 | 1.00E+04 | 1.00E+05 | 1.00E+04 | 1.00E+05 |
|  | C13 | 1.00E+03 | 1.00E+03 | 1.00E+05 | 1.00E+05 | 1.00E+05 | 1.00E+05 |
|  | Mean | 4.00E+03 | 7.00E+02 | 3.70E+05 | 1.00E+05 | 7.00E+04 | 7.00E+04 |
|  | SD | 5.20E+03 | 5.20E+02 | 5.47E+05 | 0.00E+00 | 5.20E+04 | 5.20E+04 |
| **RBMRNA-404**  **（20μg）** | D11 | 1.00E+03 | 1.00E+03 | 1.00E+05 | 1.00E+05 | 1.00E+05 | 1.00E+04 |
|  | D12 | 1.00E+03 | 1.00E+03 | 1.00E+06 | 1.00E+05 | 1.00E+05 | 1.00E+05 |
|  | D13 | 1.00E+03 | 1.00E+03 | 1.00E+05 | 1.00E+05 | 1.00E+05 | 1.00E+04 |
|  | Mean | 1.00E+03 | 1.00E+03 | 4.00E+05 | 1.00E+05 | 1.00E+05 | 4.00E+04 |
|  | SD | 0.00E+00 | 0.00E+00 | 5.20E+05 | 0.00E+00 | 0.00E+00 | 5.20E+04 |

**TableS2.** **IgG titers against Wuhan SARS-CoV-2 spike protein following immunization with the RBMRNA-405.**

| **Group** | **Sample** | **iD14** | **iD21** | **iD28** | **iD32** | **iD52** | **iD66** |
| --- | --- | --- | --- | --- | --- | --- | --- |
| **RBMRNA-405（5μg）** | E11 | 1.00E+04 | 1.00E+03 | 1.00E+05 | 1.00E+05 | 1.00E+05 | 1.00E+05 |
|  | E12 | 1.00E+05 | 1.00E+04 | 1.00E+05 | 1.00E+05 | 1.00E+05 | 1.00E+05 |
|  | E13 | 1.00E+03 | 1.00E+06 | 1.00E+05 | 1.00E+05 | 1.00E+05 | 1.00E+04 |
|  | Mean | 3.70E+04 | 3.37E+05 | 1.00E+05 | 1.00E+05 | 1.00E+05 | 7.00E+04 |
|  | SD | 5.47E+04 | 5.74E+05 | 0.00E+00 | 0.00E+00 | 0.00E+00 | 5.20E+04 |
| **RBMRNA-405（20μg）** | F11 | 1.00E+04 | 1.00E+04 | 1.00E+06 | 1.00E+05 | 1.00E+05 | 1.00E+05 |
|  | F12 | 1.00E+04 | 1.00E+04 | 1.00E+06 | 1.00E+06 | 1.00E+06 | 1.00E+05 |
|  | F13 | 1.00E+04 | 1.00E+05 | 1.00E+06 | 1.00E+05 | 1.00E+05 | 1.00E+05 |
|  | Mean | 1.00E+04 | 4.00E+04 | 1.00E+06 | 4.00E+05 | 4.00E+05 | 1.00E+05 |
|  | SD | 0.00E+00 | 5.20E+04 | 0.00E+00 | 5.20E+05 | 5.20E+05 | 0.00E+00 |

**TableS3.** **Mutations of SARS-Cov2 spike variants in Pseudovirus neutralization assay.**

| **Variants** | **Mutations in spike protein** | **Genebank accession NO.** |
| --- | --- | --- |
| Wuhan-Hu-1(Wild type) | N/A | 43740568 |
| Beta(B.1.351)* | L18F,D80A, L241-,L242-,A243-,R246I,K417N,E484K,N501Y,D614G,A701V | [ON322586.1](https://www.ncbi.nlm.nih.gov/nucleotide/ON322586.1?report=genbank&log$=nucltop&blast_rank=1&RID=911W4J8M013) |
| Gamma(P.1) | L18F,T20N,P26S,D138Y,R190S,K417T,E484K,N501Y,D614G,H655Y,T1027I,V1176F | ON471228.1 |
| Alpha(B.1.1.7) | H69-,V70-,Y144-,N501Y,A570D,D614G,P681H,T716I,S982A,D1118H | [ON442267.1](https://www.ncbi.nlm.nih.gov/nucleotide/ON442267.1?report=genbank&log$=nuclalign&blast_rank=1&RID=8YMW6YKT016) |
| Delta(B.1.617.2) | T19R,G142D，E156-,F157-,R158G,A222V,L452R,T478K,D614G,P681R,D950N | ON220436.1 |
| Omicron(BA.1) | A67V,H69-,V70-,T95I,G142-,V143-,Y144-,Y145D,N211-,L212I,ins214EPE,G339D,S371L,S373P,S375F,K417N,N440K,G446S,S477N,T478K,E484A,Q493R,G496S,Q498R,N501Y,Y505H,T547K,D614G,H655Y,N679K,P681H,N764K,D796Y,N856K,Q954H,N969K,L981F | OM287553.1 |
| Omicron(BA.2) | T19I，L24-，P25-，P26-，A27S，G142D，V213G，G339D，S371F，S373P，S375F，T376A，D405N，R408S，K417N，N440K，S477N，T478K，E484A，Q493R，Q498R，N501Y，Y505H，D614G，H655Y，N679K，P681H，N764K，D796Y，Q954H，N969K | ON526784.1 |
| Omicron(BA.4) | V3G，T19I，L24-，P25-，P26-，A27S，H69-，V70-，G142D，V213G，G339D，S371F，S373P，S375F，T376A，D405N，R408S，K417N，N440K，L452R，S477N，T478K，E484A，F486V， Q498R，N501Y，Y505H，D614G，H655Y，N679K，P681H，N764K，D796Y，Q954H，N969K | [OP614580.1](https://www.ncbi.nlm.nih.gov/nucleotide/OP614580.1?report=genbank&log$=nuclalign&blast_rank=1&RID=N4V5D795013) |
| Omicron(BA.5) | T19I，L24-，P25-，P26-，A27S，H69-，V70-，G142D，V213G，G339D，S371F，S373P，S375F，T376A，D405N，R408S，K417N，N440K，L452R，S477N，T478K，E484A，F486V，Q498R，N501Y，Y505H，D614G，H655Y，N679K，P681H，N764K，D796Y，Q954H，N969K | [OP658627.1](https://www.ncbi.nlm.nih.gov/nucleotide/OP658627.1?report=genbank&log$=nuclalign&blast_rank=1&RID=N4TZ0F08016) |

* Beta(B.1.351) pseudovirus spike protein harbors the L18F mutation in comparison with [ON322586.1](https://www.ncbi.nlm.nih.gov/nucleotide/ON322586.1?report=genbank&log$=nucltop&blast_rank=1&RID=911W4J8M013).
